# Collisions of Cortical Microtubules with Membrane Associated Myosin VIII Tail

**DOI:** 10.3390/cells11010145

**Published:** 2022-01-03

**Authors:** Sefi Bar-Sinai, Eduard Belausov, Vikas Dwivedi, Einat Sadot

**Affiliations:** The Institute of Plant Sciences, Volcani Institute, ARO, Rishon LeZion 7528809, Israel; sefi.barsinai@gmail.com (S.B.-S.); eddy@volcani.agri.gov.il (E.B.); dwivedivikas1989@gmail.com (V.D.)

**Keywords:** microtubules, myosin VIII, collisions

## Abstract

The distribution of myosin VIII ATM1 tail in association with the plasma membrane is often observed in coordination with that of cortical microtubules (MTs). The prevailing hypothesis is that coordination between the organization of cortical MTs and proteins in the membrane results from the inhibition of free lateral diffusion of the proteins by barriers formed by MTs. Since the positioning of myosin VIII tail in the membrane is relatively stable, we ask: can it affect the organization of MTs? Myosin VIII ATM1 tail co-localized with remorin 6.6, the position of which in the plasma membrane is also relatively stable. Overexpression of myosin VIII ATM1 tail led to a larger fraction of MTs with a lower rate of orientation dispersion. In addition, collisions between MTs and cortical structures labeled by ATM1 tail or remorin 6.6 were observed. Collisions between EB1 labeled MTs and ATM1 tail clusters led to four possible outcomes: 1—Passage of MTs through the cluster; 2—Decreased elongation rate; 3—Disengagement from the membrane followed by a change in direction; and 4—retraction. EB1 tracks became straighter in the presence of ATM1 tail. Taken together, collisions of MTs with ATM1 tail labeled structures can contribute to their coordinated organization.

## 1. Introduction

The plant MTs cytoskeleton is organized into two main structures: during cell division, it forms the cell-division apparatus, which is sequentially developed from the preprophase band, to the spindle and then the phragmoplast; during interphase, MTs form parallel arrays under the plasma membrane [1,2,3,4]. The latter are termed cortical MTs. Cortical MTs remain tethered to the plasma membrane along their entire length during interphase [5,6]. The retention of MTs at the cortex prevents their free lateral sliding and sinking deep into the cytoplasm. Their stable positioning at the cortex is important for the cortical MTs’ role in directing the cellulose synthase complex (CSC) to entry sites in the plasma membrane [7,8], and in forming tracks for CSC trajectories in the plasma membrane while cellulose is deposited outside the cell [9]. The attachment of cortical MTs to the plasma membrane restricts the regulation of their organization to their intrinsic machinery of nucleation, polymerization, depolymerization and bundling [6,10]. It has been shown that encounters between MTs affect their fate. When the plus-end of a MT encounters another cortical MT, it will generally co-align with it if the angle is less than 40°, and either cross over or undergo a catastrophe at the site of the encounter if the angle is more than 40° [6,11,12]. In addition, it has been shown that severing at crossover points contributes to MTs reorientation [13,14]. Some proteins were shown to tether cortical MTs to the plasma membrane. Phospholipase D was proposed as a candidate protein [15]; however, later, conflicting data regarding this possibility were obtained [2]. Another protein is the companion of cellulose synthase 1 [16]. 

An additional view of the interaction of cortical MTs with the plasma membrane milieu has been discussed. Cortical MTs have been shown to restrict protein diffusion in the plasma membrane by forming “fences” [17,18]. Under the microscope, this is often seen as black slits in the distribution pattern of membrane associated proteins exactly where the cortical MTs reside [17,18]. Importantly, specificity was demonstrated in this interaction when MTs restricted the localization of active ROP11-MIDD1 complex in the plasma membrane but not that of ROP11 alone [19]. A model of dynamic membrane organization composed of the membrane skeleton (actin) tethered by pickets (transmembrane-domain proteins) that restrict protein diffusion is well-described in animal cells [20], and has consequences on signaling by promoting protein–protein interactions or clustering [21]. Cortical MTs might play a similar role in plants.

Unconventional class VIII myosins are plant-specific actin motor proteins that localize to the plasma membrane, cell plates and plasmodesmata [22,23,24,25,26,27,28,29,30] and are involved in targeting viral proteins to the plasmodesmata [31,32]. However, the precise function of myosin VIII is not clear. Based on colocalization data, it was assumed that myosin VIII ATM1 is involved in endocytosis and ER-membrane contact sites [23]. Detailed enzymological characterization suggests its function in maintaining cortical tension [24,33], rather than powering rapid movement [24,34]. Interestingly, myosin VIII from moss binds the plus-end of phragmoplast MTs and directs their expansion specifically to the cell-division zone in the mother cell wall [25]. In addition, myosin VIII plays a role in the organization of MTs at the growing tip of moss cells [35].

We noticed that when the tail of myosin VIII ATM1 is expressed, its membrane associated distribution is often found in coordination with that of cortical MTs. Since very little lateral movement of membrane associated ATM1 tail clusters was observed, we explored the possibility that it has an effect on the organization of cortical MTs. We used the IQ-tail domain of myosin VIII ATM1 [22,23], because it is easily expressed and forms clusters associated with the plasma membrane.

## 2. Materials and Methods

### 2.1. Plants, Plasmids and Transient Expression

*Nicotiana benthamiana* and *Arabidopsis thaliana* plants were grown in a controlled growth room at 23 °C under 16/8 h light/dark conditions.

The plasmid encoding the ATM1 tail (ATM1^IQ-tail^) mCherry–ATM1^IQ-tail^ domain (At3g19960) is described in [23]. The plasmid encoding EB1b (At5g62500) was prepared as follows: cDNA was isolated by RT-PCR using the primer pair 5′CCGCTCGAGAACATGGCGACGAACATTGGG3′ and 5′GGAATTCAGTTTGGGTCTCTGCAGC3′, and ligated upstream to YFP and a bridge of 4 × Gly between XhoI and EcoRI in the plasmid pART7. The expression cassette, including the 35S promoter, was then cut by NotI and ligated into the binary vector pART27. pART27-CWLP-GFP was kindly provided by Aviah Zilberstein [36], in which we replaced GFP with mCherry. The constructs of remorins were kindly provided by Thomas Ott [37]. Seeds of Arabidopsis expressing RFP-TUA5 were from [8]. For expression of remorin 6.6 in RFP-TUA5 plants, its cDNA was isolated by PCR using the following primers: FW GCGGTACCATGGATACCTTAATCAAGCA, Rev GCGGATCCTCAGAAACAGCATGCATTTC and ligated KpnI-BamHI downstream to GFP and a 10XAla bridge in pART7. The whole expression cassette including 35S promoter was then ligated by NotI in the pML-BART plasmid. The three truncations of remorin 6.6 (aa)1–231, 232–276, and 277–343 were amplified by PCR and fused downstream to GFP as described above. All DNA fragments were fully sequenced. The plasmids were transformed into *Agrobacterium* GV3101, and transient expression was performed as previously described [22]. Transformation to Arabidopsis was performed as previously described by Clough and Bent [38].

### 2.2. Microscopy and Image Analysis

Confocal microscopy was performed as follows: an Olympus Fluoview 500 was used to acquire time-lapse movies with frames taken every 2.77 s. For these, PlanApo 60 × /1.0 Olympus water immersion objective was used, and the following filter sets: for YFP, 515 nm excitation and BA535–565 emission filter; for RFP, 543 nm excitation and BA560IF emission filter. The other microscope used was a Leica SP8 equipped with HyD detectors and HC PL APO CS 63 × /1.2 water immersion objective (Leica, Wetzlar, Germany). For EB1 movies, image series of 50 frames were acquired, using a scan field of 512 × 512 pixels and 1 sec time frame intervals. For tubulin labeled MTs dynamics, image series of 50 frames were acquired, using a scan field of 512 × 512 pixels and 2 sec time frame intervals. For the 3D images and Z axis visualization field of 1024 × 1024 pixels with line average of 2 and 7–10 optical section with 0.356 μm Z- step size were acquired. Three-dimensional reconstruction and tilting were done using the SP8 software. Imaging was performed using an OPSL 488 laser for YFP excitation with 500–540 nm emission range, and an OPSL 552 laser for RFP with 565–600 nm emission light detection. For FM4-64 staining excitation was at 488 nm and emission 610–650 nm. Imaris was used for image analyses (Bitplane, Oxford Instruments). Spots were created using Spot Creation Wizard with different spot sizes. EB1b tracks were created using the Imaris Track module. For MTs orientation, infiltration was performed into one side of the leaf, while the other side remained as a control for MTs orientation. For MTs orientation quantification the plugin directionality of ImageJ was used. The plugin computes a histogram indicating the amount of structures in a given direction. A Gaussian curve is fitted to the highest pick. The statistics generated by the plugin was used to compare the uniformity of MTs directions in 25–40 cells of each treatment by plotting the “Dispersion” (the standard deviation of the Gaussian) against the “Amount” (the sum of the histogram from center − std to center + std, divided by the total sum of the histogram) which refers to the amount of structures with close orientation. Prior to kymographs creation, time laps movies (50 frames, 2 sec intervals) were corrected for lateral drift using StackReg (Transformation: Rigid Body) Fiji plugin. A line of 10 μm was drawn passing through few ATM1^IQ-tail^ bodies and kymograph was created via KymographBuilder Fiji plugin. Maximal lateral displacement from initial position of ATM1^IQ-tail^ spots was determined by the simulation. For statistics, the GraphPad Prism 9 software was used.

### 2.3. The Simulation

A simulation tool was developed using Python script, which provided data as well as animation that helped focus on selected tracks. Track and spot statistical data from Imaris were used to draw the EB1 tracks (yellow lines or white lines for tracks that encountered collisions) and ATM1^IQ-tail^ spots (red circles or white circles for spots that encountered collisions). The simulation tool also enabled focusing on one selected track by using a different color and line width. In addition to statistical data from Imaris, the simulation enabled us to provide the following information: Collision distance—the distance between the border of the ATM1^IQ-tail^ spot and the center of the EB1 signal for each pair of EB1 signal and ATM1^IQ-tail^ spot (colliding pair) in all frames. The border of the ATM1^IQ-tail^ spots was estimated by using the “different spot diameters” option in Imaris. Whenever the distance was below the ATM1^IQ-tail^ spot radius plus a certain value (a configuration parameter usually chosen to be ≤100 nm according to [39,40]), we considered it to be a collision, and the colliding pair was highlighted. The simulation provided the following measurements: (1) The number of collisions per EB1 track and (2) per ATM1^IQ-tail^ spot, and (3) the percentage of colliding tracks or spots out of total in the field captured. (4) The speed of the EB1 tracks near the collision (near collision duration was set by two configuration parameters—the number of frames before and after the collision time frame, here used one frame before and three after). (5) The difference between the speed and straightness of the EB1 track before and after the collision compared to the whole track. (6) The number and percentage of EB1 tracks that went through acceleration or deceleration or not affected after colliding, compared to the average speed of the whole track. (7) Percent acceleration events that were associated with a decrease in straightness. (8) Maximal lateral displacement from initial position of ATM1IQ-tail spots.

The simulation data was written to a csv file for further processing. The animation could be saved as a gif or mpg file.

The following is a Python code snippet that we used to calculate collisions between colliding pairs:

# pos is a matrix with EB1 and ATM1 spot coordinates from Imaris statistics.

# D is a matrix with distances between all pairs of EB1 and ATM1 spots.

D = squareform(pdist(pos))

# Get indices of close spot pairs (diam is the matrix of ATM1 diameters).

ind1, ind2 = np.where(D < diam)

# Avoid duplicate close pairs.

unique = ind1 < ind2

ind1 = ind1[unique]

ind2 = ind2[unique]

## 3. Results

### 3.1. Coordinated Positioning of Cortical MTs and ATM1^IQ-tail^

**Figure 1:**
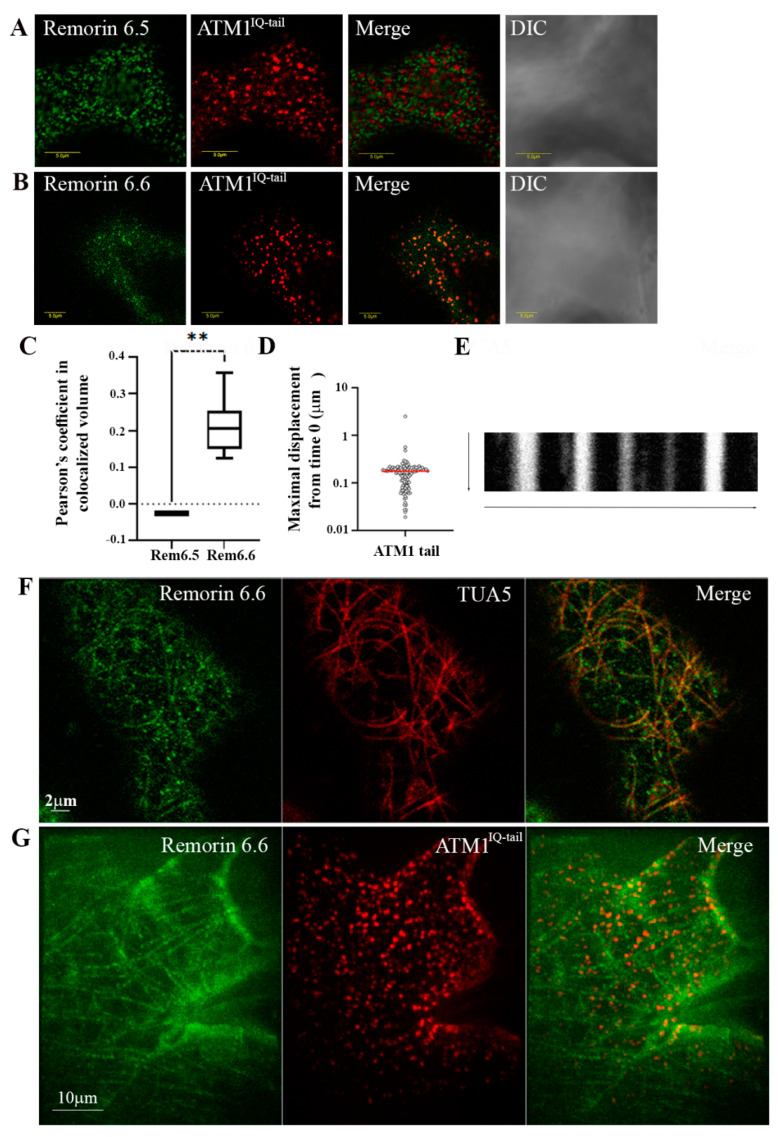
Myosin VIII ATM1^IQ-tail^ colocalizes with remorin 6.6 labeled structures. (**A**) Negative colocalization with remorin 6.5. (**B**) Colocalization with remorin 6.6. (**C**) Pearson’s coefficient analysis of colocalization. Differences were analyzed by *t*-test ** *p* < 0.01. (**D**) Kymograph showing lateral stability of ATM1^IQ-tail^; Y axis—50 frames, 2 sec intervals, X axis—10 μm. (**E**) Maximal displacement from position in time 0, of each ATM1^IQ-tail^ spot, during 50 frames, 2 sec intervals in 3 cells. (**F**) Partial alignment of GFP-remorin 6.6 with MTs labeled with mCherry-Tubulin α5 in *A. thaliana*. (**G**) No pronounced colocalization of mCherry-ATM1^IQ-tail^ with GFP-remorin 6.6 along MTs, in *N. benthmiana*.

As mentioned, ATM1^IQ-tail^ is associated with the plasma membrane [22,23]. To check the nature of ATM1^IQ-tail^ cortical clusters, we compared them to membrane proteins from the remorin family [37]. Partial colocalization was found with remorin 6.6, but not with remorins 1.2, 1.3, 1.4, 3.1, 3.2, 4.1, 4.2, 5.1, 6.1, 6.2, 6.4 (not shown), or remorin 6.5 (Figure 1A–C), suggesting that ATM1 associates with remorin 6.6. To identify the potential domain of remorin 6.6 regulating the colocalization with ATM1, the following fragments from remorin 6.6 were prepared and fused to GFP at their N terminus: the variable N terminus (aa 1–231), the coiled-coil domain (aa 232–276), and the typical remorin C terminus (aa 277–343) (Figure S1A). The coiled-coil domain was separated from the C-terminal tail by the existence of a coiled-coil domain in myosin VIII, and it was therefore necessary to rule out a possible nonspecific interaction between the two sticky domains. Transient expression of these constructs in *N. benthamiana* showed GFP in the cytoplasm (Figure S1B–D), indicating that neither one of the two C-terminal peptides alone is sufficient to mediate plasma membrane binding of the fusion protein. However, the finding that the C-terminal 20 aa of remorin 6.6 are not able to anchor GFP to the membrane are in agreement with previously published data [41]. When GFP–remorin 6.6^1–231^, GFP–remorin 6.6^232–276^ and GFP–remorin 6.6^277–343^ deletion fragments were coexpressed with mCherry–ATM1^IQ-tail^ (Figure S1E–G), GFP–remorin 6.6^1–231^ was recruited to the mCherry–ATM1^IQ-tail^ puncta (Figure S1E), showing colocalization with a slightly better Pearson’s value than the full-length remorin 6.6 (Figure S1H), and indicating an ATM1–remorin 6.6 binding site in this domain. Similar to remorin 6.6 [37], lateral mobility of ATM1^IQ-tail^ is limited as shown by a kymograph (Figure 1D) and by calculating the maximal displacement from position at time 0 of all spots during 50 frames, showing the average of 0.177 ± 0.22 μm (Figure 1E). GFP-remorin 6.6 forms clusters associated with the plasma membrane and often, but not always, is seen along MTs [37] and Figure 1F. Interestingly, colocalization of ATM1^IQ-tail^ and remorin 6.6 is most notable in the clusters, but not along MTs that are labeled only by GFP-remorin 6.6 (Figure 1G). The mechanism that controls the transition of remorin 6.6 to MTs and whether myosin VIII plays a role in it are still unclear.

**Figure 2:**
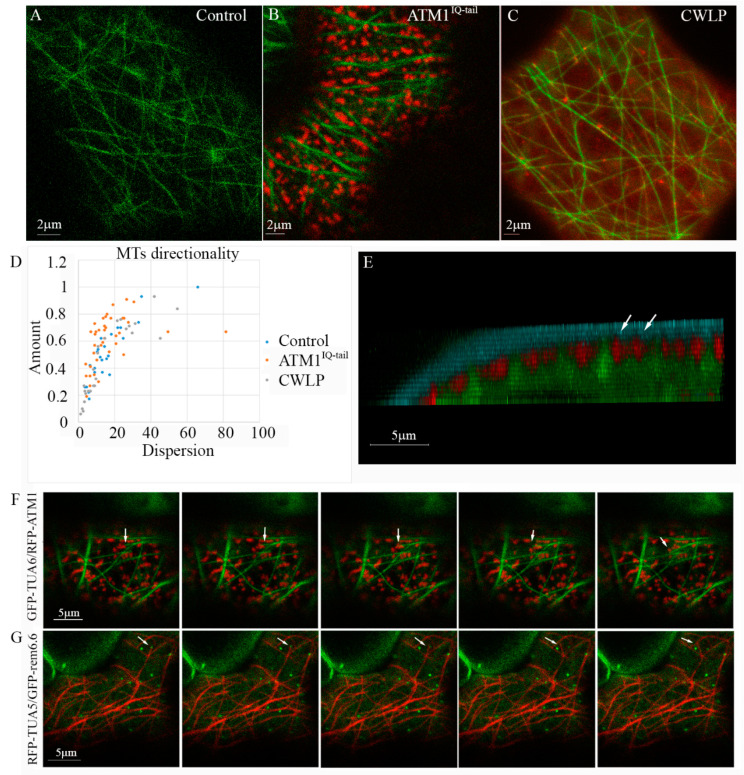
Coordinated organization of ATM1^IQ-tail^ and cortical MTs. 35S-GFP-TUA6 expressing *N. benthamiana* plants (**A**) were infiltrated with 35S-mCherry-ATM1^IQ-tail^ (**B**) or CWPL-RFP (**C**). Confocal microscopy was performed 48 h after *Agrobacterium* infiltration, for imaging the abaxial side of epidermal cells. (**D**) MTs orientations were determined by the directionality plugin of ImageJ in 25–40 cells. Amount refers to the fraction of MTs and dispersion to the distribution of different angles. (**E**) Z-axis view of a *N. benthamiana* epidermal cell labeled with FM4-64 (blue), ATM1^IQ-tail^ (red-arrows), MTs (green). (**F**) Five frames (2 sec intervals) from Movie S2 showing a shift (arrows) in MT orientation after a collision with ATM1^IQ-tail^ cluster in *N. benthamiana* epidermal cells expressing GFP-TUA6 and mCherry ATM1 ^IQ-tail^. (**G**) Five frames (2 sec intervals) corresponding to Movie S3 showing a shift (arrows) of orientation of mCherry-TUA5 labeled MT after a collision with GFP-remorin 6.6 labeled structure in *A. thaliana* epidermal cell.

In this work, we addressed the question whether clusters of membrane associated ATM1^IQ-tail^ affect MTs positioning in the cortex of epidermal cells. For this 35S-mCherry-ATM1^IQ-tail^ [22,23] was transiently expressed in 35S-GFP-TUA6 stably expressing *N. benthamiana* plants [42], and its distribution in the cells was followed by live cell imaging microscopy (Figure 2A–D). As previously described for other proteins [17,18,19], the MTs were frequently localized in straight empty spaces (slits) formed between the membrane zones that were enriched with ATM1^IQ-tail^ (Figure 2B). Strikingly, when MTs orientation was quantified it was found that a larger fraction of MTs (“Amount”) had similar orientation (lower “Dispersion”) in the presence of overexpressed ATM1^IQ-tail^ than in control cells (Figure 2A,B,D). Suggesting that excess ATM1^IQ-tail^ had an effect on the organization of cortical MTs in these cells. To determine whether this also happen in the presence of another membrane associated protein we used Cell Wall Linker Protein CWLP-mCherry [36]. It was found that the distribution of MTs’ orientations in the presence of excess CWLP was similar to control (Figure 2C,D). To get a better picture of the relative positioning under the membrane, we examined cells expressing GFP-TUA6 and mCherry-ATM1^IQ-tail^, stained with the membrane dye FM4-64. Figure 2E shows 3D tilted image, which demonstrates the three stained constituents in the Z-axis. It shows that the signal of ATM1^IQ-tail^ is beneath the FM4-64 stain, and MTs are positioned a little further below it, between the labeled mCherry-ATM1^IQ-tail^ spots (See also Movie S1 for a broader view of a cell).

In order to monitor MTs behavior near ATM1^IQ-tail^ clusters, we have acquired time lapse movies using the GFP-TUA6 stable expressing *N. benthamiana* plants transiently expressing mCherry-ATM1^IQ-tail^. While very little lateral movement of ATM1^IQ-tail^ clusters was detected (Figure 1D,E and Movies S2, S5–S8), a detachment of MT from the membrane and rapid deviation in the direction of its elongation was observed after a collision with ATM1^IQ-tail^ cluster (Figure 2F and Movie S2). Further, it was examined if MTs collide with remorin 6.6 clusters in *A. thaliana*. MTs dynamics was followed in mCherry-TUA5 plants expressing GFP-remorin 6.6. Figure 2G and Movie S3 show rapid change in MT orientation following a collision with a remorin 6.6-labeled structure.

**Figure 3:**
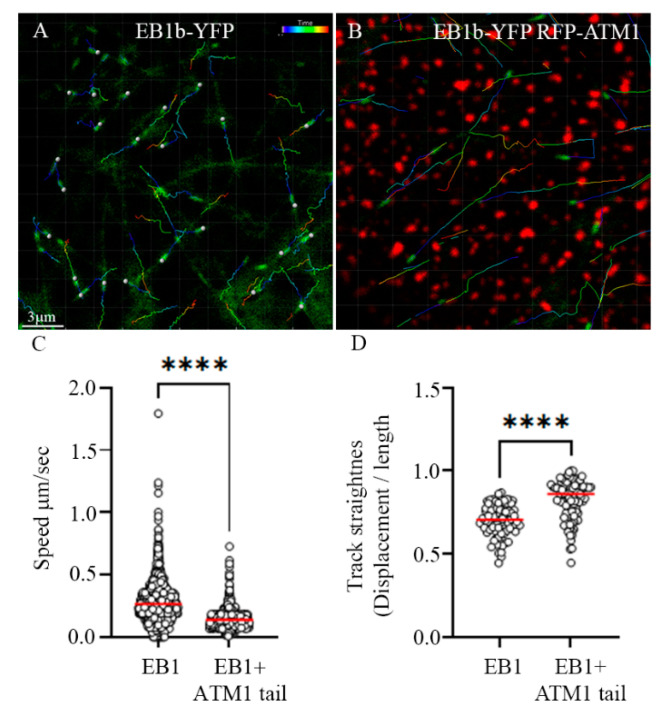
Changes in elongation rate and track straightness of MTs in the presence of overexpression of ATM1^IQ-tail^. EB1b-YFP was expressed alone (**A**), or in the presence of mCherry-ATM1^IQ-tail^ (**B**) in *N. benthamiana* leaves. Time-lapse movies were acquired (50 frames, frame/1 s) and image analysis was done using Imaris. Scale bar = 3 μm. (**C**) EB1b speed was calculated from all tracks every 1 s. (**D**) Track straightness was calculated by dividing the displacement by the total length. Calculations were performed for ten independent cells of each expression combination. The color code show the age of the track; start = blue, end = red. Differences were determined by *t*-test **** *p* < 0.0001.

In order to closely examine the interaction of ATM1^IQ-tail^ clusters with MTs, EB1b-YFP was co-expressed alone or with mCherry-ATM1^IQ-tail^ in *N. benthamiana* and time-lapse movies were acquired (Figure 3 and Movie S4 for EB1 alone and Movie S5 for EB1 + ATM1^IQ-tail^). EB1 is a plus end MT binding protein which marks the elongating tips and promote polymerization [5]. Details of EB1b-YFP speed and straightness of the tracks were extracted with the image analysis software Imaris. While the average speed of EB1b-YFP when expressed alone was 0.266 ± 0.152 μm/sec that in the presence of mCherry-ATM1^IQ-tail^ was reduced to 0.139 ± 0.068 μm/sec (measurements of all MTs during 50 frames in ten cells for each expression combination) (Figure 3C). It is interesting to note that the measured EB1b-YFP speeds were faster than those generally measured for MTs labeled by GFP tubulin [6]; however, these are known differences previously reported in Arabidopsis and animal cells [43,44]. We then looked at the straightness of EB1b-YFP tracks by determining the ratio between displacement and total length. Track straightness was lowest when EB1b-YFP was expressed alone 0.7 ± 0.09, whereas when it was co-expressed with RFP-ATM1^IQ-tail^, the tracks straightened slightly, but significantly, 0.83 ± 0.1 (Figure 3D). Taken together, this suggested that the presence and organization of ATM1^IQ-tail^ can affect MTs straightness and average elongation rate.

### 3.2. Collision of MTs with ATM1^IQ-tail^ Clusters Can Result in Cross Over, Reduced Elongation Rate, Detachment and Reorientation or Retraction

**Figure 4:**
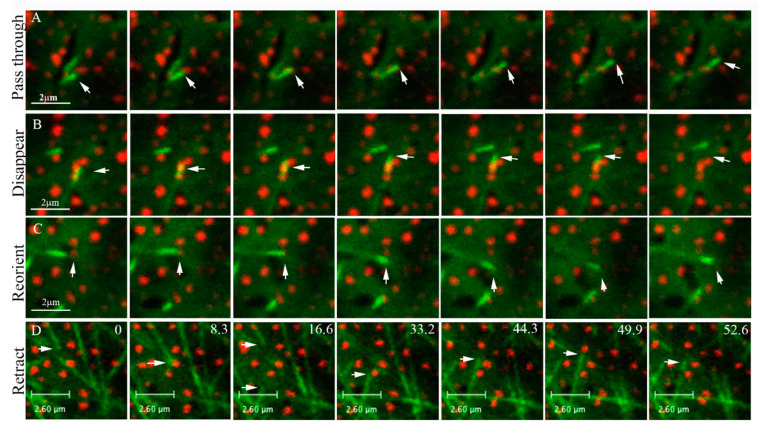
The four scenarios of collisions between MTs and ATM1^IQ-tail^ clusters. Seven frames, 1 s apart, in (**A**–**C**) (corresponding to Movie S5), several seconds apart as depicted by numbers in (**D**) (corresponding to Movie S7), show the following: (**A**) a MT passing through a spot, (**B**) a MT overlapping a spot, pausing and then disappearing; (**C**) a MT colliding with a spot and changing direction; (**D**) two MTs retracting after collision.

To characterize and quantify the collisions with clusters of ATM1^IQ-tail^, again EB1b-YFP and mCherry-ATM1^IQ-tail^ were co-expressed in *N. benthamiana* and time-lapse movies were acquired. It was found that EB1b mainly moves between ATM1^IQ-tail^ spots (see Movie S5 and 4 dimension image acquisition in Movie S6), probably along pre-existing bundles; however, occasionally, there were collisions between EB1b and ATM1^IQ-tail^. We could define four resultant scenarios for such collisions: (1) the MT passes through an ATM1^IQ-tail^ cluster and continues elongating (Figure 4A arrows and Movie S5); (2) the MT pauses at an ATM1^IQ-tail^ cluster and then disappears (Figure 4B arrows and Movie S5), either because it leaves the focal plane or because EB1b dissociates from the MTs; (3) the MT collides with an ATM1^IQ-tail^ cluster, detaches from the membrane, waves from side to side and eventually continues to elongate in another direction (Figure 4C, arrows, and Movie S5); (4) the MT retracts or undergoes a catastrophe upon a collision with an ATM1^IQ-tail^ cluster (Figure 4D arrows and Movie S7).

**Figure 5:**
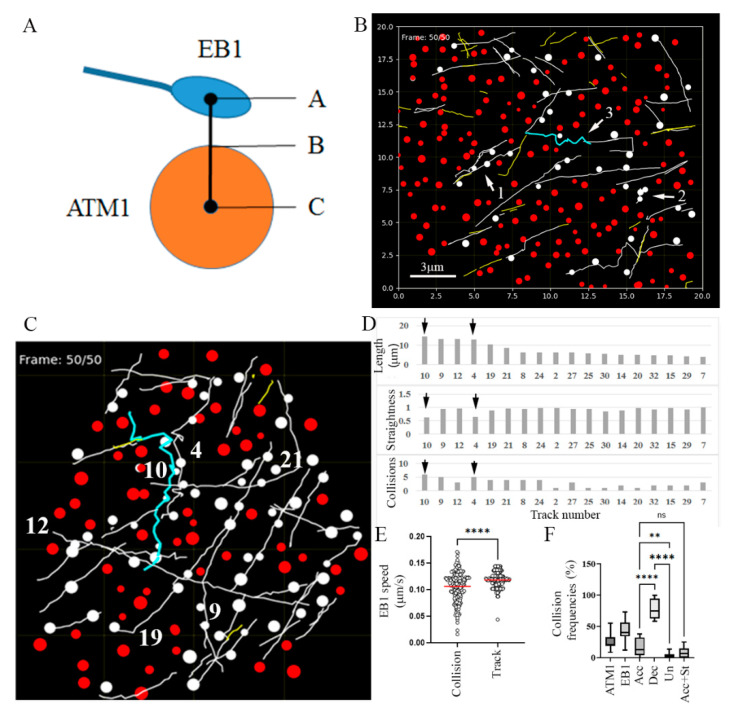
A software program developed in Python that simulates MTs–ATM1^IQ-tail^ collisions. (**A**) Scheme showing the concept of the simulation: coordinates of the positioning of each ATM1^IQ-tail^ cluster or EB1b spot were extracted from Imaris. A collision was counted when the distance from A to B was ≤100 nm. (**B**) Snapshot of animation based on Movie S5. Spots of ATM1^IQ-tail^ that experience MTs collision and the colliding track turned white. A selected track was colored. Arrows indicate three of the four scenarios: (1) pass through, (2) pause and disappear, (3) wave and change direction. Scale bar = 3 μm. (**C**–**E**) A comparison of all tracks by a single cell analysis. (**C**) Snapshot of the last frame of the animation (Movies S8 and S9) showing, in white, tracks and spots that experienced collisions. The numbers show the six longest tracks. Track 10 which experienced maximum collisions is colored. (**D**) Summary of length, straightness and number of collisions for all tracks above 5 μm in length. Arrows show tracks 10 and 4 as being the least straight. (**E**) EB1b speed in frames −1 to +3 around every collision compared to the average speed along the whole track. The graph shows results from the cell presented in panel (**C**). Differences were determined by *t*-test *p* < 0.0001. (**F**) Collisions frequencies calculated in 20 cells; ATM1-percent ATM1^IQ-tail^ spots experienced collisions, EB1-percent colliding tracks, Acc-percent tracks with accelerated speed after a collision. Dec-percent tracks with decelerated speed after a collision. Un-percent tracks with unaffected speed. Acc + str-percent tracks with accelerated speed associated with decreased straightness. ** *p* < 0.01, **** *p* < 0.0001 one way ANOVA. ns = not significant.

To quantify the collision events, we developed a simulation software that identified those events according to a minimum defined distance between the border of the ATM1^IQ-tail^ spots and the center of the EB1b signal (Figure 5A). Defining a distance of 100 nm or less from the border of ATM1^IQ-tail^ cluster to the center of the EB1b signal provided a simulation of collisions that was analogous to that observed visually. The border of the ATM1^IQ-tail^ spots was estimated by the size of the spot diameters attributed by Imaris to each ATM1^IQ-tail^ spot. We first animated Movie S5 and confirmed that scenarios 1–3 were simulated correctly (Figure 5B). Note that the zigzag pattern shown in the simulation result (blue track in Figure 5B) depicts the waviness of the growing tip, but does not show the actual pattern of the MT itself.

We then asked if the waviness of a track is correlated to the number of collisions it has experienced. Figure 5C–E follow all tracks longer than 5 µm in a cell (see also Movies S8 and S9). Track numbers 10 (14.4 μm) and 4 (12.9 μm), which collided six and five times, respectively, exhibited a zigzag-like pattern with a low degree of straightness (0.63 and 0.65, respectively), which seemed to result from the extra waviness of EB1b. On the other hand, tracks 9 (13.1 μm) and 12 (13.1 μm), which experienced five and three collisions, respectively, exhibited straightness of 0.94 and 0.96, respectively (Figure 5D). Taken together, this suggested that tracks 10 and 4 might not elongate along existing bundles, but break through a new route full of obstacles. It might also suggest that at least part of the MT detachment from the plasma membrane and waviness is a result of a collision with a cluster of ATM1^IQ-tail^. However, it is also suggested that not all collisions result in MTs detachment or loss of straightness. We then compared the average speed of EB1b-YFP movement along one frame before a collision and three frames after it, and compared this to the average speed along the whole track, for all collisions, in this cell (Figure 5E). A significant reduction in the average EB1b-YFP speed was found to be associated with the collision events. This suggested that the reduction in average EB1b-YFP speed found in the cells co-expressing ATM1^IQ-tail^ (Figure 3) was a result of the collisions. Furthermore, an increase in the variability among the different EB1 speeds near a collision was observed with much slower and much higher rates than the variability observed in the whole track. We also used the simulation to determine the frequencies of collision (Figure 5F). By analyzing 20 cells, it was found that 25.6% ± 11.3% of ATM1^IQ-tail^ spots and 42.9% ± 16.2% of EB1 tracks experienced collisions. In addition, out of the colliding MTs, in 17.8% ± 14.0% the speed was accelerated after a collision, in 78.8% ± 15.8% it was decelerated and in 3.4% ± 4.0% the speed was unaffected. In 8.4% ± 8.3%, the acceleration in speed was associated with a decrease in straightness which might reflect those events described in Figure 2F,G, Figure 4C and Figure 5C,D in which, MTs collide, detach from the membrane, wave and change direction.

## 4. Discussion

We show that cortical MTs can collide with ATM1^IQ-tail^ clusters associated with the membrane, an event that can lead in most cases to a decreased rate of elongation and seldom to detachment from the membrane and change in orientation. MTs can also change orientation after a collision with a remorin 6.6 labeled cluster. ATM1^IQ-tail^ partially co-localizes with remorin 6.6, a family member of the remorins that are associated with plant plasma membrane nanodomains [37,45,46,47,48] and have a role in biotic and biotic stresses [49]. Colocalization of ATM1^IQ-tail^ with remorin 6.6 raises the possibility that myosin VIII has an affinity for unique structures in the membrane. Another family of proteins that may be related to the system described here is the family of IQD calmodulin binding protein, which includes membrane proteins and microtubule-binding proteins [50]. The pattern of membrane localization of IQD24 and IQD25 is reminiscent of that of remorin 6.6 and similarly their lateral mobility is low [50]. Thus, it would be interesting to check whether microtubules collide with IQD24 and IQD25 membrane associated clusters and whether their organization beneath the membrane is coordinated.

Taken together, the plant plasma membrane is sub-compartmentalized into consecutive dense networks of proteins [37] with different densities and sizes of domains protruding into the cytoplasm. In addition, different proteins can perturb the shape of the plasma membrane [51], suggesting that the inner surface area of the membrane in the vicinity of the elongating MTs is complex and dynamic. Thus, the waviness of MTs plus ends, observed in time-lapse movies [6], might be the result of collisions with invisible different membrane associated structures. Interestingly, more frequent MTs detachment from the plasma membrane was observed in a *clasp-1* mutant compared to wild-type *Arabidopsis* plants [52]. In the mutant *clasp-1* cells, the detached plus-end of the growing cortical MTs underwent several waving movements from side to side before reattaching to the plasma membrane in a different orientation [52]. A mathematical model showed that membrane detachment in clasp-1 enabled MTs to entrain more readily than undergo a catastrophe upon high angle encounters [53]. Therefore, it may be concluded that occasional detachments increase the degree of freedom of MTs to change their organization, and are under the regulation of CLASP.

MTs-ATM1^IQ-tail^ collisions had consequences on the overall coordinated organization of MTs arrays in *N. benthamiana* epidermal cells. The process seems to contribute to a straighter cortical MTs and a uniform array, correlated to the organization of ATM1^IQ-tail^ associated with the membrane. These conclusions are based on quantitative image analysis and a newly developed simulation that facilitated the quantification of collisions and their consequences on MTs dynamics. Interestingly, whereas here, MTs straightness seemed to be affected by the cross-talk with ATM1^IQ-tail^, in general, the actual orientation of MTs is known to be affected by physical mechanisms [54,55]. It has been shown in vitro that MTs can sense compression or extension derived deformation of an elastic surface [56]. Therefore, if the organization of membrane associated proteins is influenced by tension or compression exerted on cells, this might be sensed by MTs. We propose that the concept revealed here of cross talk between MTs and membrane associated structures might reflect a more general situation in plant cells. Full length ATM1 expression in *A. thaliana* has been done in the past and the protein has been shown to concentrate in dots at the cell cortex of protoplasts and cotyledon epidermal cells [24]. In addition, moss myosin VIII localized as cortical particles both in moss and in BY2 cells [25]. We were unable to express the full length ATM1 fused to GFP in normally growing Arabidopsis plants. Therefore, the IQ-tail fragment of ATM1 is used here as a proxy of the whole protein, but might behave differently. Nevertheless, it has been used as an effective tool for exposing the phenomenon of microtubule collisions with membrane associated proteins, supported here also by demonstrating the case with full length remorin 6.6.

To the best of our knowledge, collisions of plant cortical MTs with clusters of plasma membrane associated structures have never been described or considered as influential events in cortical MTs organization, and this finding is therefore novel.

Collisions of plant cortical MTs with membrane associated structures are reminiscent of MTs targeting or passing through focal adhesions in mammalian cells, as previously described [39,57,58,59]. Focal adhesions are protein complexes that tether actin fibers to the membrane via transmembrane-domain proteins from the integrin family, which in turn are anchored to the extracellular matrix [60]. In animal cells, MTs are likely to be targeted to focal adhesions along actin filaments [39]. In addition, MTs plus-end binding proteins such as EB1 and CLASP have been found to be associated with active integrin complexes [61]. How plant MTs target myosin VIII ATM1^IQ-tail^ spots and whether MTs transiently interact with these clusters still need to be determined. In this respect, it should be mentioned that although myosin VIII-MTs interaction was shown in a different plant system [25,35] no persistent colocalization between the two was observed in *N. benthamiana* leaf epidermal cells.

## 5. Conclusions

Taken together our data suggest that not only MTs form a corral for the diffusion of membrane associated proteins but some membrane associated proteins might also form physical obstacles for, or even transient biological interactors with MTs plus-ends, such that their reciprocal interaction can eventually dictate membrane compartmentalization and coordinated MTs organization.

## Data Availability

All data supporting the findings are described in this article. Additional details about the simulation, as well as all biological reagents are available from the corresponding author Einat Sadot upon request.

## Supplementary Materials

The following are available online at https://www.mdpi.com/article/10.3390/cells11010145/s1, Figure S1: The interaction between ATM1^IQ-tail^ and remorin 6.6. Movie S1: Z axis rotation showing relative poisoning of the membrane (blue) MTs (green) and ATM1^IQ-tail^ (red). Movie S2: GFP-TUA6 labeled MT colliding with ATM1^IQ-tail^ cluster in *N. benthamiana.* Movie S3: mCherry-TUA5 labeled MT colliding with remorin 6.6 cluster in *A.thaliana.* Movie S4: YFP-EB1b tracks. Movie S5: YFP-EB1b and mCherry-ATM1^IQ-tail^; collisions. Movie S6: YFP-EB1b and mCherry-ATM1^IQ-tail^; 4D. Movie S7: YFP-EB1b and mCherry-ATM1^IQ-tail^; retraction upon collision. Movie S8: YFP-EB1b and mCherry-ATM1^IQ-tail^; collisions 2. Movie S9: YFP-EB1b and mCherry-ATM1^IQ-tail^; animation of Movie S8.
