# Supplementary material for: Collisions of Cortical Microtubules with Membrane Associated Myosin VIII Tail
Source: Cells. 2022 Jan 3;11(1):145. doi: 10.3390/cells11010145 (PMC8750215; doi:10.3390/cells11010145)
Supplement: Supplementary file 1 [file cells-11-00145-s001.zip › cells-1518804-SM/Figure S1.pdf]

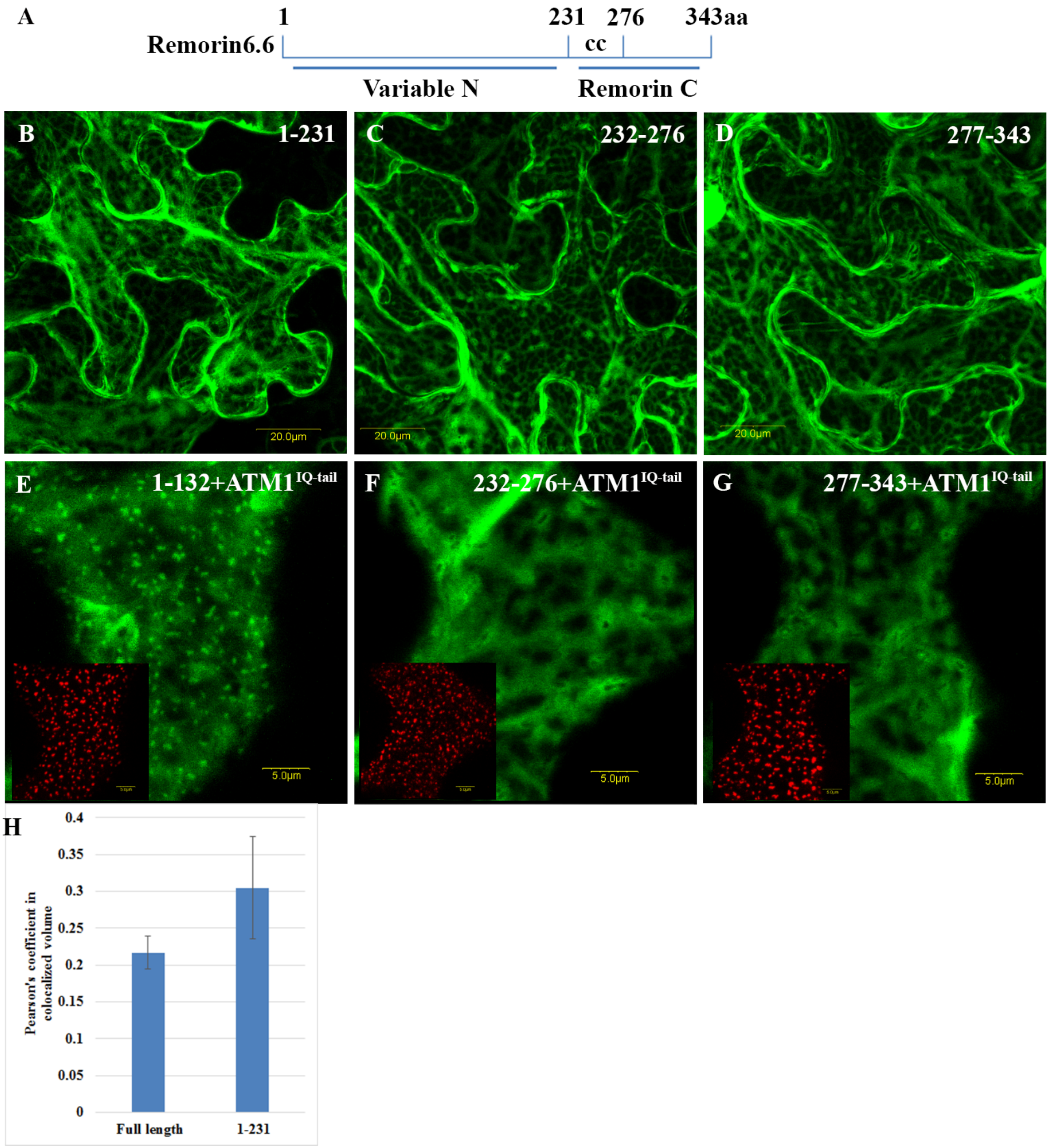

Figure S1. The interaction between ATM1$^{\text{IQ-tail}}$ and remorin 6.6. (**A**) a scheme showing the fragments of remorin 6.6. (**B–D**). The expression of these fragments fused to GFP. (**E–G**). Coexpression with mCherry-ATM1$^{\text{IQ-tail}}$ showing the recruitment of remorin 6.6 $^{1\text{-}231}$ to the plasma membrane by mCherry-ATM1$^{\text{IQ-tail}}$. (**H**). Pearson coefficient analysis confirms the colocalization of GFP-remorin 6.6 $^{1\text{-}231}$ compared to the full length remorin6.6 with mCherry-ATM1$^{\text{IQ-tail}}$.
