# Supplementary figures and images for: Collisions of Cortical Microtubules with Membrane Associated Myosin VIII Tail
Source: Cells. 2022 Jan 3;11(1):145. doi: 10.3390/cells11010145 (PMC8750215; doi:10.3390/cells11010145)

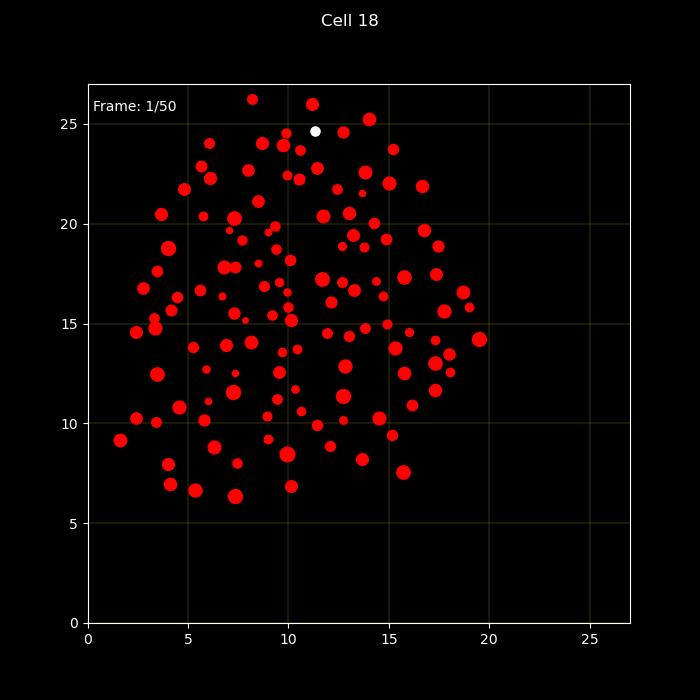

Supplement: Supplementary file 1 [file cells-11-00145-s001.zip › cells-1518804-SM/movie S9.gif]
